# Surviving but not thriving: inconsistent responses of zooxanthellate jellyfish polyps to ocean warming and future UV-B scenarios

**DOI:** 10.1038/srep28859

**Published:** 2016-07-04

**Authors:** Shannon G. Klein, Kylie A. Pitt, Anthony R. Carroll

**Affiliations:** 1 Australian Rivers Institute–Coasts and Estuaries, Griffith School of Environment, Gold Coast Campus, Griffith University, QLD 4222, Australia; 2 Environmental Futures Research Institute, Griffith School of Environment, Gold Coast Campus, Griffith University, QLD 4222, Australia

## Abstract

Complex changes to UV radiation at the Earth’s surface are occurring concurrently with ocean warming. Despite few empirical tests, jellyfish are hypothesised to be increasing in some parts of the world because they are robust to environmental stressors. Here we examine the effects of UV-B and ocean warming projections on zooxanthellate jellyfish polyps. We exposed *Cassiopea* sp. polyps to three levels of UV-B (future-low (1.43 Wm^2^), current (1.60 Wm^2^), future-high (1.77 Wm^2^)) and two levels of temperature (current-day (25 °C) and future (28 °C)) over 6 weeks. The intensity of UV-B was varied throughout the day to mimic diel variation in UV-B irradiance. Polyp survival, asexual reproduction and YII were measured. In the current and future-high UV-B treatments, more polyps were produced in 25 °C than 28 °C. This pattern, however, was reversed under future-low UV-B conditions, where more polyps were produced at 28 °C. YII was highest under current summer conditions and future conditions of low UV-B and increased temperature. YII, however, was reduced under high UV-B conditions but was further reduced with warming. Our results suggest that although *Cassiopea* polyps may survive elevated UV-B and warming conditions, they are unlikely to thrive. If, however, UV-B radiation decreases then ocean warming may facilitate increases in *Cassiopea* populations.

Increased levels of ozone depleting substances (ODSs) in the Earth’s atmosphere partially eroded the ozone layer between the 1960s and 1990s, resulting in increased levels of ultraviolet B (UVB) radiation^1^. Since the 1990s, the levels of ODSs in the Earth’s atmosphere have slowly reduced, which has halted the depletion of total ozone^2^ and since 2000 the total ozone has increased by ~1%^3^,^4^,^5^, but see^6^.

Although ozone recovery could decrease levels of UV-B radiation at the Earth’s surface, other climate system factors such as ozone chemistry and climate change may strongly influence ozone recovery and make future UV-B radiation levels challenging to model^7^. Recent projections for human sunburn risk, the erythemally weighted irradiance (UV_ery_) (comprised of UV-A and UV-B, but see^8^), suggest that ozone recovery could decrease UV_ery_ by up to 10% outside polar regions by 2100. However, projected improvements in air quality, reductions of aerosols and decreased cloud cover could also increase UV_ery_ by 10%^6^,^9^. The intensity of UV radiation is not uniform across the Earth’s surface. Some locations in the southern hemisphere, such as New Zealand, experience higher UV index (UVI) irradiance than locations in the northern hemisphere of similar latitudes^10^.

Excessive UV-B radiation can have several adverse effects on marine biota^11^. Increased UV-B radiation can reduce fecundity, slow development and cause mortality of several life history stages of marine organisms^12^,^13^,^14^. UV-B radiation also inhibits light-induced electron transport in photosystem II (PSII) in marine plants^15^. Zooxanthellae, the microalgal symbionts of corals and other invertebrates, may be particularly sensitive to UV radiation because zooxanthellae often exist at temperatures close to their thermal threshold and exposure to elevated UV reduces temperature bleaching thresholds^16^. Negative effects on zooxanthellae, caused by excessive UV radiation, can have several flow-on effects to the host by reducing carbon translocation and damaging host tissues^16^,^17^. In some cases, severe photoinhibition causes zooxanthellae to be expelled from the host^16^,^18^. Thus, animals and plants may be affected by elevated UV-B radiation.

Changes to UV-B radiation are occurring concurrently with ocean warming. Of the studies that empirically examined the effects of elevated UV and increased temperature on marine biota (e.g. refs 19,20), few studies considered the effects on symbioses involving zooxanthellae. The few studies that have examined the interactive effects of elevated temperature and UV radiation on zooxanthellate symbioses consistently demonstrate either additive or synergistic responses to the dual stressors^16^,^17^,^21^,^22^. For example, exposure only to high temperature decreased densities of zooxanthellae in the gorgonian, *Eunicea tourneforti*. Although high UV-B did not, on its own, affect zooxanthellae densities, *E. tourneforti* exposed to high temperature and high UV-B simultaneously, experienced 100% mortality^22^. These results suggest that warming temperatures may further increase the susceptibility of zooxanthellae to UV radiation. Thus, it is vital to determine the potential interactive effects of future UV-B levels with future ocean warming projections to obtain a realistic understanding of how biota are likely to respond to changing ocean conditions.

Most studies of the effects of UV-B radiation on marine biota investigate the effects of exposure to constant levels of UV-B radiation over several hours^23^ or days^17^,^19^. In marine systems, however, UV-B irradiance fluctuates throughout the day and may interact with tidal cycles. For example, low tides that occur in the middle of the day, when UV-B is most intense, simultaneously expose shallow marine biota to high UV radiation and mid-day temperature extremes^24^. Studies that mimic diel changes in UV-B radiation in combination with other co-occurring environmental stressors will provide the most realistic understanding of the effects of UV-B radiation in the natural environment.

Despite few empirical tests, jellyfish populations are hypothesised to be increasing in some parts of the world because they are considered to be robust to a range of environmental stressors^25^. Recent research, however, suggests jellyfish may not be immune to environmental stressors^26^,^27^ and changes to UV radiation may adversely affect jellyfish populations. Indeed the freshwater hydrozoan, *Limnocnida tanganyicae*, died when exposed to elevated UV for only one hour and avoidance of UV was postulated as the reason why this species migrates to deeper waters during the day^28^. Species of jellyfish that host zooxanthellae, however, must occur in shallow waters to expose their zooxanthellae to light for photosynthesis. Whilst zooxanthellate medusae can alter their depth to optimise exposure to light, those that occur in shallow coastal lagoons may have limited ability to move vertically. Moreover, their zooxanthellate polyps (the asexual, benthic stage of the life history) are sessile and may be unable to avoid UV-B extremes.

Studies on the effects of climate change on jellyfish are limited, but in scyphozoan jellyfish, elevated temperature can increase rates of asexual reproduction of polyps^29^,^30^, strobilation^31^,^32^ and growth^33^. Some scyphozoans, such as *Cassiopea* spp., form symbioses with one or more clades of zooxanthellae^34^. Zooxanthellae often exist close to their thermal thresholds, and *Cassiopea* spp. commonly form symbioses with thermally sensitive *Symbiodinium spp.* (e.g. *Symbiodinium microadriaticum*)^35^,^36^,^37^. Prolonged exposure to thermal stress can trigger some cnidarian hosts to expel zooxanthellae and damage host tissues^38^. Although the combined effects of UV and temperature have not been studied on jellyfish, most studies of the effects of UV and temperature on other cnidarian symbioses demonstrate either additive or synergistic responses to the combined stressors^16^,^17^,^22^,^39^. Consequently, zooxanthellate jellyfish may be more susceptible to the combined effects of UV and elevated temperature than non-zooxanthellate species.

*Cassiopea* spp. typically occur in tropical and subtropical waters worldwide where they sometimes form noticeable blooms^40^,^41^. They have dense concentrations of zooxanthellae in their oral arms and reside upside-down on the benthos. *Cassiopea* sp. medusae can persist year-round but medusae populations are restocked during the summer months from asexual reproduction of benthic polyps^42^. Larvae of *Cassiopea* sp. are non-symbiotic but once they metamorphose into primary polyps, they acquire zooxanthellae via horizontal transmission^34^,^43^. Zooxanthellate polyps of *Cassiopea* sp. have been observed in areas of high illumination and so are sometimes exposed to intense UV-B radiation (Klein, personal observation).

The overall aim of this study was to assess the potential interactive effects of future UV-B radiation scenarios (both increasing and decreasing) and ocean warming projections on polyps of *Cassiopea* sp. Specifically, we hypothesised that *Cassiopea* sp. polyps exposed to increased UV-B and increased temperature conditions separately would exhibit negative effects on asexual reproduction and photochemical efficiency. Given that other cnidarian symbioses have exhibited either additive or synergistic responses to these combined stressors^16^,^17^,^21^,^22^, we hypothesised that when exposed to both increased UV-B and increased temperature simultaneously, the effects would be more severe than those of the individual stressors. Under reduced UV-B conditions, however, we hypothesised that there would be no effect on asexual reproduction and photochemical efficiency and, therefore, when exposed to both reduced UV-B and increased temperature, *Cassiopea* sp. polyps would only respond negatively to increased temperature.

## Methods

### Experimental approach

Polyps of *Cassiopea* sp. were sampled from a 10 m^3^ display tank at Underwater World, Sunshine Coast, Queensland in September 2013. Once collected, all polyps were acclimated to laboratory conditions by exposing them to 25 °C for 8 weeks prior to the commencement of the experiment and were fed newly hatched *Artemia* sp. nauplii daily. During acclimation, polyps were exposed to 14 hours of natural sunlight to optimise photosynthesis of zooxanthellae. *Cassiopea* sp. polyps used in this study were not identified to species because of morphological similarities within the *Cassiopea* genus^44^.

*Cassiopea* sp. polyps were exposed to two orthogonal factors: UV-B radiation (three levels: future-low UV-B (1.43 Wm^−2^), current-day UV-B (1.6 Wm^−2^), and future-high UV-B (1.77 Wm^−2^)) and temperature (two levels: current-day (25 °C) and future (28 °C)). Current-day UV-B levels, diel fluctuations in UV-B and current temperature conditions were based on field measurements taken during summer on the 9^th^ January, 2015, when low tide was at midday at Saltwater Creek, southeast Queensland (SEQ), Australia (−27.90 °S, 153.37 °E) (Supplementary Fig. S1) where populations of *Cassiopea* sp. commonly occur. UV-B measurements were taken underwater at the same depth that *Cassiopea* sp. polyps occur in the field. To accurately mimic the scenario that future UV-B levels could decrease by up to 10% because of ozone recovery^6^,^9^, a 10% decrease was determined relative to current-day UV-B levels measured in the field (future-low UV-B). To mimic the scenario that future UV-B levels could increase by 10% because of improvements in air quality and decreased cloud cover^6^,^9^, a 10% increase was determined relative to current-day conditions (future-high UV-B). Future temperatures were based on summer temperatures predicted to occur in SEQ waters based on the RCP 6.0 scenario for the yr. 2100^45^.

Four polyps were transferred using a toothpick into plastic petri dishes that were weighted with stainless steel weights. Five replicate 1L glass aquaria, each containing four petri dishes (i.e. a total of sixteen polyps per replicate aquarium), were randomly allocated to each UV-B and temperature combination (i.e. N = 30). In each replicate aquarium, two of four petri dishes were allocated for chlorophyll fluorescence measurements and the others were used for polyp reproduction measurements. Two petri dishes were allocated to each dependent variable to prevent overcrowding of polyps and for ease of cleaning. The number of polyps in each replicate aquarium was summed across reproduction dishes for statistical analyses (i.e. the unit of replication was the total number of polyps in the two reproduction dishes for each replicate aquarium). The experiment was done in a controlled temperature laboratory with the ambient temperature set at 22 °C. Temperatures within aquaria were raised to their required levels (current-day (25 °C) and future (28 °C)) using aquarium heaters. Each replicate aquarium was partially submerged within a 5L water bath and aerators were used to circulate the water and maintain an even distribution of heat. Temperatures were maintained to within 0.4 °C of desired levels. Polyps in all aquaria were gradually introduced into their respective treatment conditions by raising the temperature at a rate of ~0.75 °C d^−1^ and by altering UV-B radiation levels from control levels (1.6 Wm^−2^) by ±25% of the desired UV-B levels for each treatment until desired conditions were reached (i.e. all polyps were slowly introduced into their respective treatments over 4 days).

Aquaria were gently aerated at the surface to maintain >6.0 mgL^−1^ of oxygen and to circulate water. The dissolved oxygen (DO) concentration of each aquarium was tested daily using an optic DO sensor (Mettler Toledo OptiOx). Plastic lids were placed loosely over each replicate aquarium with a header space of ~1.5 cm to minimise evaporation. Temperature was recorded daily in each aquarium using a thermometer and salinity was measured using a conductivity-salinity metre (TPS salinity-conductivity metre MC-84). The temperature of each water bath was monitored daily and adjusted if required. Approximately 70% of the water in each aquarium was exchanged with fresh 10 μm filtered seawater daily and petri dishes were gently wiped to remove excess algae and biofilm. The experiment ran for 37 days.

**Figure 1:**
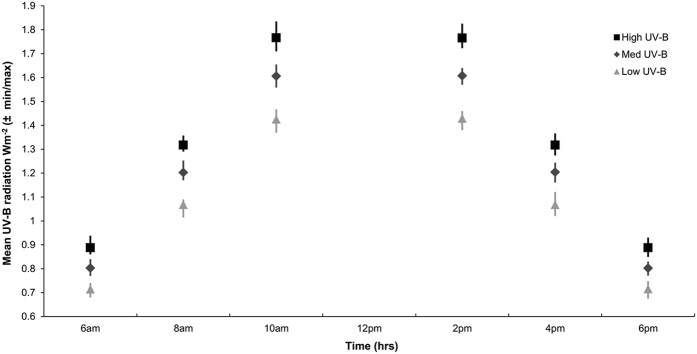
Mean (±minimum/maximum) UV-B radiation (Wm^−2^) levels for current and future UV-B conditions predicted for SEQ throughout the 37 day experiment. UV-B radiation levels were averaged across temperature levels. UV-B radiation levels were recorded at two-hourly intervals, except for between the hours of 10am and 2pm, where UV-B levels were held constant.

All aquaria were exposed to 12 hours of light (UV-B and PAR) per day to accurately mimic diel patterns during summer. Aquarium lights were used to imitate the natural solar spectrum, with the wave crest located in the blue spectrum (400–500 nm) to optimise photosynthesis of zooxanthellae. To achieve the desired UV-B conditions aquaria were exposed to UV-B lights (Phillips UV-B Broadband TL 40 W/ 12 RS; bandwidth spectrum: 290–315 nm, peak: 302 nm) and covered with polyvinyl chloride (PVC) to filter out short wavelengths that do not reach the Earth’s surface (UV-C, 100–280 nm) (as used by^19^). To accurately mimic diel patterns of UV-B radiation, the height of each UV-B-light was manually adjusted every 2 hours throughout the day, except for between 10am and 2pm when UV-B radiation levels were held constant. UV-B levels were adjusted by ±25% increments of the desired UV-B levels for each treatment to mimic diel fluctuations (see Fig. 1). UV-B intensity was measured six times per day, when UV-B intensity was manipulated, using a waterproofed UVB radiometer (UVP, model: UVX). The UV-B radiometer measured unweighted intensity between 280–340 nm. The UV-B radiometer was submerged in each aquarium and UV-B intensity was measured at the same depth as the polyps throughout the experiment.

### Data collection

Three response variables were measured: survival, asexual reproduction rates and photochemical efficiency. Every third day, all polyp dishes were removed from aquaria and the number and developmental stage (i.e. scyphistoma (polyp stage), budding of planuloid buds, or strobila (partial strobilation)) of individuals was recorded using a dissecting microscope (see^43^,^46^). If necessary, a compound microscope (at low magnification) was used to inspect polyps for newly formed planuloids. Only once the planuloid buds metamorphosed into sessile polyps were they recorded as polyps and used as the dependent variable. Each replicate aquarium was thoroughly checked for newly formed polyps that did not settle on the polyp dishes. Polyps were checked for survival and recorded as dead when polyp tissues started to disintegrate. Prior to the commencement of the experiment, and at weekly intervals throughout the experiment, photochemical efficiency was measured using a Phyto-Pulse amplitude modulator (Phyto-PAM). Prior to fluorescence measurements, one polyp from each replicate aquarium was transferred into a plastic vial. All polyps were dark-adapted for 30 minutes prior to fluorescence measurements. The Phyto-PAM measured several photosynthetic parameters of symbiotic zooxanthellae under low light conditions. Repetitive measurements of the Chl *a* fluorescence parameters yielded a measure of the minimum (F_0_) and maximum fluorescence yield (F_m_) from which the effective quantum yield (YII) was determined^47^. After all measurements were recorded, polyps were returned to the aquaria and fed by introducing ca. 50 newly hatched *Artemia* sp. nauplii into each petri dish. After 2 hours, the water in each aquarium was partially replenished to remove excess nauplii.

### Statistical analyses

The dependent variables (number of polyps and YII) in all treatments were analysed using repeated measures linear mixed models (LMMs) in SPSS^48^. The three fixed factors were: UV-B radiation (1.77 Wm^−2^, 1.6 Wm^−2^, and 1.43 Wm^−2^), temperature (28 °C and 25 °C), and time (days 1–37 (number of polyps) or weeks 1–6 (YII)), which was the repeated measure. A range of models were investigated to assess the model of best fit by comparing various goodness-of-fit statistics (−2 log likelihood, AIC, and BIC). For all analyses, data were checked for normality and homoscedasticity using standardised residual and Q-Q plots and, if required, data were ln(*x* + 1) transformed. If significant differences were found, estimated marginal means were used to determine which means differed.

## Results

### Maintenance of UV-B conditions and water chemistry

UV-B radiation levels for each treatment accurately mimicked diel patterns for current day conditions and ca. 2100 future predictions^45^ (Fig. 1). The mean (±1 SE) temperature levels were maintained at 25.0 °C (±0.04) and 28.1 °C (±0.01). During the experiment salinity was maintained at 36.1 ppt (±0.91).

### Survival and asexual reproduction

**Figure 2:**
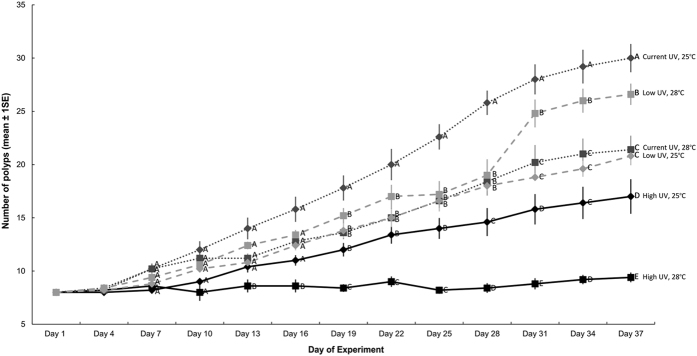
Mean (±1SE) number of polyps recorded at three-day intervals during the 37 day experiment at current and future temperature levels (Days 1–37, n = 30). Letters next to data points on Day 37 indicate similarities (e.g. AA) or difference (e.g. AB) between temperature treatments within each UV-B level, as determined by estimated marginal means.

All polyps of *Cassiopea* sp. survived throughout the experiment, except for four polyps in the high UV-B, high temperature treatment. Over time, the number of polyps increased in all treatments. The temporal variation in the number of polyps differed among UV-B treatments, but patterns were not consistent among temperature treatments, resulting in a significant Time × UV-B × Temperature interaction (Table 1; Fig. 2). The rate of increase was greatest in the current UV-B, low temperature treatment (i.e. current day conditions). From Day 19 onwards, the number of polyps in this treatment exceeded those in all other treatments (Fig. 2). Relative to current-day conditions, at the end of the experiment, high UV and high temperature alone resulted in 44% and 29% fewer polyps, respectively. Exposure to both high UV and high temperature, however, resulted in 69% fewer polyps suggesting an additive interaction (Fig. 2). Within the high UV-B level, 45% fewer polyps were produced in the high temperature treatment than the current temperature treatment at the end of the experiment. Polyps in the high temperature, high UV-B treatment began to bud by Day 7, but two polyps died at Day 10 and again at Day 25. Rates of asexual reproduction in this treatment remained slow and at the end of the experiment the average number of polyps had increased by only one (Fig. 2). In the current and high UV treatments more polyps were produced in the 25 °C than the 28 °C treatments. This pattern was reversed, however, in the low UV treatment where 22% more polyps were produced in the 28 °C treatment than the 25 °C treatment (Fig. 2). The low UV, high temperature treatment produced 11% fewer polyps than current day conditions. Exposure to low UV and high temperature alone, however, resulted in 31% and 29% fewer polyps than current day conditions, respectively, suggesting an antagonistic interaction between low UV and high temperature (Fig. 2). Strobilation was not observed during the experiment.

### Effective quantum yield

**Figure 3:**
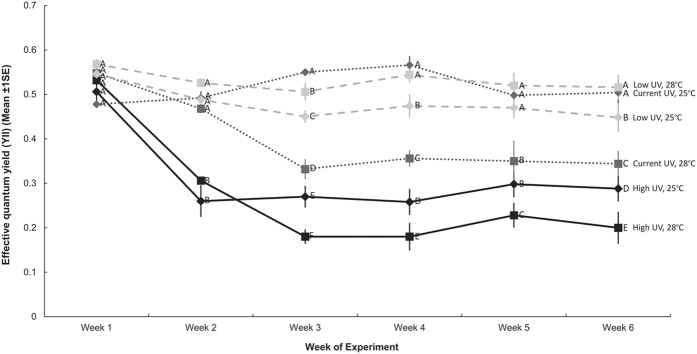
Effective quantum yield (mean ±1SE) recorded at weekly intervals during the 37 day experiment (Weeks 1–6, n = 30). Letters next to data points on Day 37 indicate similarities (e.g. AA) or difference (e.g. AB) between temperature treatments within each UV-B level, as determined by estimated marginal means.

The temporal variation of YII values among UV-B treatments was not consistent across the two temperature treatments, resulting in the Time × UV-B × Temperature interaction (Fig. 3, Table 2). At Week 1, there was no difference in YII values among treatments and in Week 2, photochemical efficiency was only reduced in polyps exposed to high UV-B. In Week 3, YII was reduced in the high UV-B treatments and in the current UV-B, high temperature treatment. The rank order of the various treatments varied slightly through time but at the end of the experiment YII was highest in the polyps in the current UV-B, low temperature treatment and the low UV-B, high temperature treatment and was lowest in the high UV-B, high temperature treatment (Fig. 3). Within the low UV-B treatments, YII was lowest in the low temperature treatment but in the current and high UV-B treatments, YII values decreased with increasing temperature.

## Discussion

The pattern of asexual reproduction supported our hypothesis that the combined effects of increased UV-B and increased temperature would be more severe than those of the individual stressors and highlights the need to study interactions between stressors. Most *Cassiopea* sp. polyps survived high UV-B conditions, but the high UV-B, high temperature treatment produced 25% fewer polyps than the low temperature treatment, suggesting an additive interaction between increased temperature and UV-B. Although warming reduced rates of asexual reproduction under current and high UV-B conditions, increased temperature enhanced rates of asexual reproduction under low UV-B conditions, suggesting an antagonistic interaction between increased temperature and low UV-B. Indeed, if future UV-B levels increase (because of improved air quality), future ocean warming predictions for the region would probably reduce polyp reproduction, suggesting that rates of asexual reproduction could be much slower in the future. If, however, future UV-B conditions are reduced by ozone recovery, future ocean warming could enhance rates of asexual reproduction and *Cassiopea* sp. polyps may continue to thrive.

The survival of organisms that have an obligate association with zooxanthellae ultimately depends on the survival of their symbionts. Photochemical efficiency of zooxanthellae was highest under current summer conditions, and, similar to the result for asexual reproduction, suggests that current environmental conditions are optimal for photosynthesis. There was, however, no significant difference between current summer conditions and the low UV-B, high temperature treatment, suggesting that if UV-B radiation levels decrease but temperature continues to increase, the symbionts of *Cassiopea* sp. will still thrive. Current UV-B, combined with warmer temperatures reduced YII of zooxanthellae, suggesting that if UV-B radiation levels remain constant but temperature continues to increase, symbionts of *Cassiopea* sp. may be adversely affected. Although elevated UV-B radiation decreased the photochemical efficiency of zooxanthellae under current temperature conditions, YII was further reduced when polyps were exposed to elevated temperature and UV-B radiation simultaneously. These data further support our observations that warmer temperatures may increase the susceptibility of *Cassiopea* sp. polyps to increased UV-B radiation.

Photochemical efficiency and asexual reproduction of polyps were similarly affected by exposure to UVB and warming. Indeed, at the end of the experiment only two treatments (low UV-B, low temperature and current UV-B, high temperature) differed in the order of their response. Similar effects on both cnidarian host tissues and their algal symbionts have been observed when exposed to increased solar radiation and thermal stress. Exposure to high solar irradiance and thermal stress caused DNA damage and apoptosis in the host coral, *Montastraea faveolata*, and also reduced photochemical efficiency and carbon fixation in its symbionts^16^. These results highlight the complexity of stress responses in symbiotic cnidarians and are consistent with the translocation of important carbon resources from zooxanthellae being tightly coupled with the productivity of the host^49^.

*Cassiopea* sp. polyps had complex responses to UV-B radiation and temperature, with variable responses to UV-B depending on the temperature tested. Although data are limited, the interactive effects of UV-B and temperature are not consistent across taxa. For example, germination was reduced at 10 °C at all UV radiation levels in the intertidal algae, *Alana marginata* and *Fucus gardneri*, but germination rates of *A. marginata* were much lower than *F. gardneri*^20^. In the current study, *Cassiopea* sp. polyps in the high UV-B treatments were exposed to a maximum UV-B irradiance of 1.77 Wm^−2^ over 37 days, whereas Hoffman *et al*.^20^ exposed the algae to a higher maximum UV-B irradiance (2.45 Wm^−2^) over a shorter exposure time (56 h), perhaps to mimic intertidal conditions at low tide. Although *A. marginata* and *F. gardneri* were exposed to more extreme UV conditions, differences in results between Hoffman *et al*.^20^ and the current study may be due to different exposure times or species-specific responses.

Our understanding of the impacts of changing environmental conditions on marine biota is hindered by the degree to which manipulative experiments can accurately mimic natural environmental conditions. Most manipulative experiments that investigate the effects of UV-radiation expose biota to continuous levels over several hours or days (e.g. ref. 16,19,50). Tidal regimes and diel fluctuations of UV radiation, however, expose sessile marine organisms to varying levels of UV-radiation, with the most extreme conditions occurring when mid-day maxima in UV radiation coincides with low tides. Hoffman *et al*.^20^ exposed *A. marginata* and *F. gardneri* to constant levels of UV-B irradiance over a 12: 12-h light: dark cycle, whereas the current study exposed *Cassiopea* sp. polyps to diel fluctuations in UV-B irradiance, yielding different responses. The only other study to investigate the effects of mid-day extremes of UV-B, exposed embryos of the intertidal gastropod, *Siphonaria australis* to the combined effects of elevated UV-B, high temperature and high salinity for 4 h each day over 5 days^19^. These conditions mimicked tidepool conditions during summer and the duration of this study probably reflected the duration of embryological development of *S. australis*. Larvae of *S. australis* experienced high mortality when exposed to the combined effects of all three stressors. In the current study, *Cassiopea* sp. polyps were exposed to diel fluctuations of UV-B radiation and elevated temperature over 37 days to mimic current and future conditions in a shallow system, from which they were unable to move. Although *Cassiopea* sp. polyps were subjected to overall longer periods of exposure to UV-B radiation, differences in results obtained by Fischer and Phillips (2014) compared to those in our study may be due to sudden changes in environmental conditions (before and after daily 4 h incubations) and the added stressor of high salinity experienced in shallow tide pool conditions. If manipulative experiments do not consider the gradual increase, mid-day maxima and gradual decrease of UV conditions experienced in the field, results may not accurately reflect how biota are likely to respond.

Our observations that *Cassiopea* sp. polyps were negatively affected by elevated UV-B are consistent with those of the only other study of the effects of UV radiation on jellyfish^28^. The freshwater hydrozoan, *L. tanganyicae* died after only 1 h exposure to high UV conditions, but were unaffected when exposed to conditions where UV wavelengths were filtered out. The more adverse effects seen on *L. tanganyicae* probably occurred because they vertically migrate to avoid areas of high illumination and, therefore, may lack photoprotective mechanisms such as energy-dependent quenching of PSII (see^51^) that zooxanthellate jellyfish may possess. Our results further support the observation that elevated UV radiation may negatively affect several life history stages and suggests that jellyfish may be susceptible to the effects of elevated UV radiation. Furthermore, our observations that elevated temperature increased the susceptibility of *Cassiopea* sp. polyps to UV-B exposure, suggests that the effects of other environmental stressors in combination with UV radiation should be considered.

The extent to which we can accurately predict how biota, such as *Cassiopea* sp., may respond to changing UV-B and temperature conditions depends partly on whether biota can acclimate to changing conditions. Marine biota have several photoprotective mechanisms that reduce damage caused by UV radiation and, therefore, may allow biota to acclimate to high UV conditions. UV absorbing compounds such as mycosporine-like amino acids (MAAs) are synthesised by many marine organisms during exposure to levels of high UV radiation^52^. MAAs are characterised by their UV-absorption maxima which extends across much of the UV spectrum and blocks harmful UV-A and UV-B wavelengths^53^. MAAs have not been found in non-zooxanthellate *Cassiopea xamachana* polyps, but have been detected in all other life history stages that have zooxanthellae (i.e. ephyrae and medusae)^54^,^55^. Although MAAs have been identified in non-symbiotic marine invertebrates^56^,^57^,^58^, MAAs are more common in microalgal-invertebrate symbioses^59^,^60^,^61^ (see^53^). These results suggest that in *C. xamachana*, it is the zooxanthellae rather than the host that produce MAAs. In this study, *Cassiopea* sp. polyps contained zooxanthellae and it is likely that the symbiosis can produce MAAs and has the potential to acclimate to high UV conditions. The effects of climate change stressors on the acquisition of zooxanthellae by polyps, however, have not been studied and climate change stressors may impair the ability of polyps to acquire zooxanthellae and, therefore, acclimate to high UV conditions. Although our results suggest zooxanthellate *Cassiopea* sp. polyps did not acclimate to high UV-B and increased temperature conditions over 37 days, longer-term experiments may more accurately determine whether biota can acclimate to elevated UV conditions.

Some researchers claim that jellyfish will thrive under future ocean warming conditions^25^,^62^. Our data suggest, however, that *Cassiopea* spp. populations will only thrive if warming coincides with decreased UV-B and that if UV-B increases then some *Cassiopea* species may decline. To more accurately predict how jellyfish, as a group, will respond to changing UV-B conditions we must now determine whether our results for *Cassiopea* are consistent with other species (zooxanthellate and non-zooxanthellate) and assess the responses of other life history stages. Predicting how marine biota, more generally, are likely to respond to changing UV-B conditions requires investigating possible interactions between other environmental stressors specific to the ecosystem being investigated and the ability of marine organisms to acclimate.

## Additional Information

**How to cite this article**: Klein, S. G. *et al*. Surviving but not thriving: inconsistent responses of zooxanthellate jellyfish polyps to ocean warming and future UV-B scenarios. *Sci. Rep.* **6**, 28859; doi: 10.1038/srep28859 (2016).

## Supplementary Material

Supplementary Information

## Figures and Tables

**Table 1:** Results of linear mixed models comparing the number of polyps between treatments during the 37 day experiment (data recorded at three-day intervals) (Days 1–37, n = 30).

Factor	Numerator df	Denominator df	F	P value
UV-B	2	35.998	59.334	<0.001
Temp	1	35.998	16.741	<0.001
UV-B × Temp	2	35.998	14.438	<0.001
Time	12	251.315	73.227	<0.001
Time × UV-B	24	251.315	6.525	<0.001
Time × Temp	12	251.315	3.509	<0.001
Time × UV-B × Temp	24	251.315	2.676	<0.001

Data were ln(*x* + 1) transformed. Df = degrees of freedom.

**Table 2:** Results of linear mixed models comparing the effective quantum yield between treatments during the 37 day experiment (data recorded at weekly intervals) (Weeks 1–6, n = 30).

Factor	Numerator df	Denominator df	F	*P* value
UV-B	2	48.349	181.702	<0.001
Temp	1	48.349	13.849	0.001
UV-B × Temp	2	48.349	25.181	<0.001
Time	5	91.258	26.605	<0.001
Time × UV-B	10	91.258	7.448	<0.001
Time × Temp	5	91.258	5.395	<0.001
Time × UV-B × Temp	10	91.258	3.054	0.002

Data were ln(*x* + 1) transformed. Df = degrees of freedom.
