# Supplementary material for: Surviving but not thriving: inconsistent responses of zooxanthellate jellyfish polyps to ocean warming and future UV-B scenarios
Source: Sci Rep. 2016 Jul 4;6:28859. doi: 10.1038/srep28859 (PMC4931449; doi:10.1038/srep28859)
Supplement: Supplementary Information [file srep28859-s1.pdf]

Surviving but not thriving: inconsistent responses of zooxanthellate jellyfish polyps to ocean warming and future UV-B scenarios

Shannon G. Klein[1*], Kylie A. Pitt[1], Anthony R. Carroll[2]

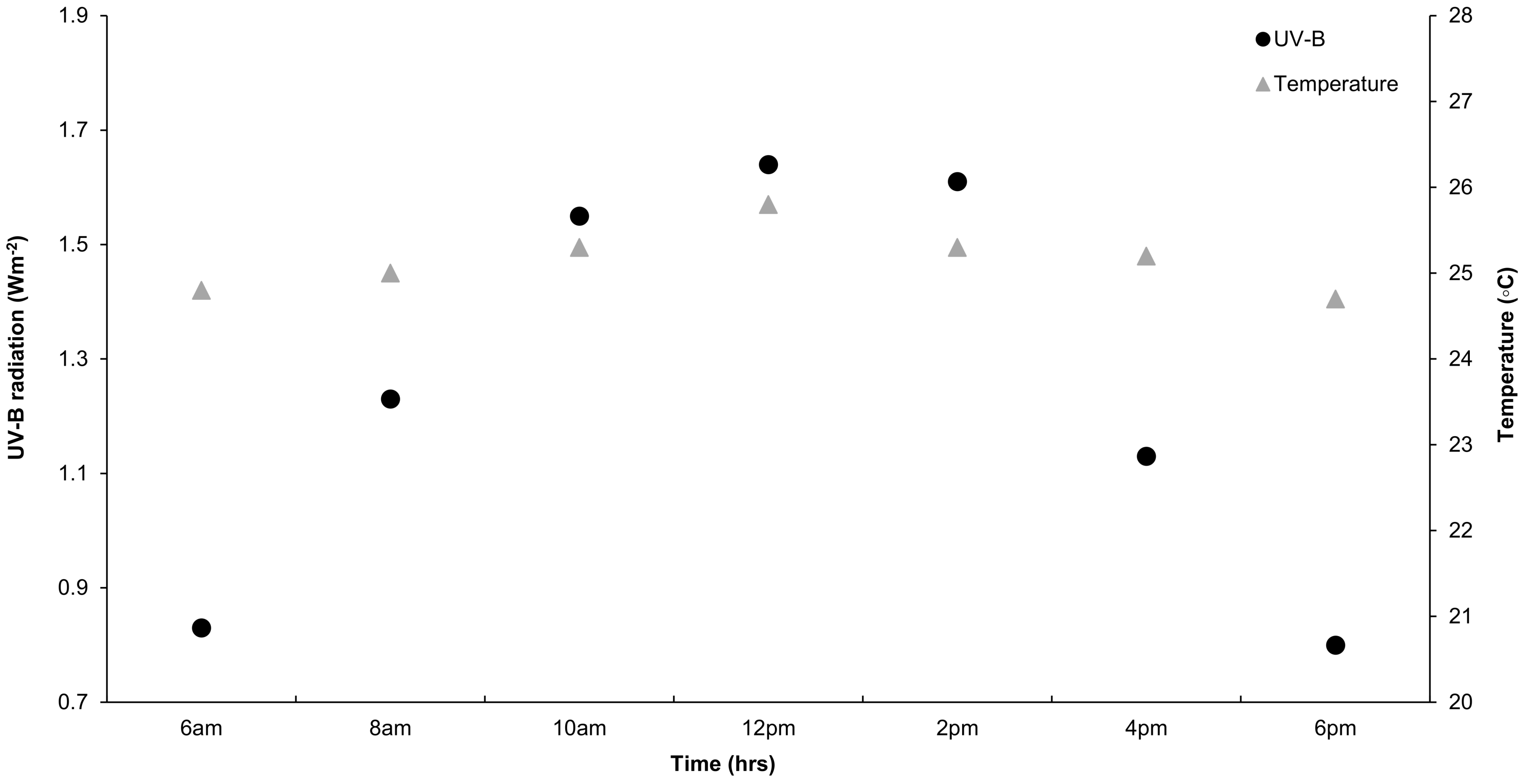

Supplementary Fig. S1 Field measurements of UV-B radiation (Wm^{-2}) and temperature (◦C) recorded on the 9th January, 2015 at Saltwater Creek, southeast Queensland (SEQ), Australia (-27.90◦S, 153.37◦E).
